# T96. A RETROSPECTIVE DATABASE STUDY OF THE RELATIONSHIP BETWEEN ALCOHOL AND CANNABIS USE AND CLINICAL MEASURES IN EARLY PHASE PSYCHOSIS

**DOI:** 10.1093/schbul/sby016.372

**Published:** 2018-04-01

**Authors:** Jacob Cookey, Philip Tibbo, Jacob McGavin, Sherry Stewart

**Affiliations:** 1 Dalhousie University; 2 Capital District Health Authority; 3 University of Toronto

## Abstract

**Background:**

Alcohol is the most commonly abused substance in Canada (18%), with cannabis use being the second most commonly abused substance (7%). People diagnosed with psychotic disorders have similar or increased risk of alcohol and cannabis use disorders compared to the general population. While there is ample data investigating the negative impact cannabis has on the development and course of psychosis, there is very limited data examining the potential role that alcohol might play. This study, therefore, investigates the pattern of alcohol and cannabis use and the clinical impact it might have in those at the early phase of psychotic illness.

**Methods:**

This is a cross-sectional, retrospective, database study of 264 patients at time of admission to the NSEPP (Nova Scotia Early Psychosis Program), in Nova Scotia, Canada. Outcome measures included the following domains: demographics/pattern of use, symptomatology, cognition and function. Four groups of patients with early phase psychosis (EPP) were analyzed according to risk level of current substance use: 1) low risk substance use (LR, n = 44), 2) moderate-high alcohol use only (AU, n = 33), 3) moderate-high cannabis use only (CU, n = 55), and 4) moderate-high combined alcohol and cannabis use (AU+CU, n = 132).

**Results:**

Between group comparisons revealed statistically significant differences in: age (with the AU group being oldest), gender (with LR and AU groups with higher % females compared to CU and CU+AU groups), Positive psychotic symptoms (with AU group having the least positive symptoms), anxiety (with AU group having most anxiety symptoms), and functioning (with AU group having higher social/occupational functioning scores).

**Discussion:**

Our findings reveal significant between group differences in a group of 264 patients at time of entry into the NSEPP. The age differences may suggest that those who develop psychosis at an older age could be more predisposed to use alcohol primarily. Alternatively, it is possible that alcohol somehow delays the onset of psychosis. The gender differences suggest that females with EPP use cannabis less commonly, and seem to choose alcohol use only if they are substance users. There are less intense positive psychotic symptoms (as measured by the positive and negative syndrome scale, PANSS) and more intense trait anxiety symptoms (measured by the state trait anxiety inventory, STAI) in the AU group which may either indicate that anxiety is worsened and positive symptoms are somehow suppressed with alcohol use, or else might reflect a subgroup in the EPP population (with less positive symptoms and more anxiety symptoms) that self-select for alcohol use. Finally, the AU group have the highest social/occupational functioning (measured with the social and occupational functioning assessment scale, SOFAS), which may indicate that alcohol facilitates social interaction/functioning; however based on existing literature, this finding more likely represents the fact that the more social someone is, the more likely they are to be exposed to alcohol in their peer group, and are therefore more likely to drink alcohol.

